# Effectiveness of mobile text reminder in improving adherence to medication, physical exercise, and quality of life in patients living with HIV: a systematic review

**DOI:** 10.1186/s12879-021-06563-0

**Published:** 2021-08-23

**Authors:** Sam Chidi Ibeneme, Sandra C. Ndukwu, Hellen Myezwa, Franklin Onyedinma Irem, Fortune Elochukwu Ezenwankwo, Adedayo Tunde Ajidahun, Amarachi D. Ezuma, Amaka Nnamani, Obinna Onodugo, Gerhard Fortwengel, Victor C. Uwakwe

**Affiliations:** 1 Department of Medical Rehabilitation, Faculty of Health Sciences, University of Nigeria, Enugu Campus, Enugu, Nigeria; 2 Department of Physiotherapy, Faculty of Health Sciences, School of Therapeutic Studies, University of the Witwatersrand, 7 York Road, Parktown, Johannesburg, 2193 South Africa; 3 Department of Physiotherapy, University of Nigeria Teaching Hospital, Ituku/Ozalla, Enugu, Nigeria; 4 Division of Exercise Science and Sports Medicine, University of Cape Town/Sports Science Institute of South Africa, Cape Town, South Africa; 5 Department of Radiation Medicine, Faculty of Medical Sciences, College of Medicine, University of Nigeria, Enugu Campus, Enugu, Nigeria; 6 University of Nigeria Centre for Clinical Trials (UNNCET), Enugu Campus, Enugu, Nigeria; 7 Department of Medicine, Faculty of Medical Sciences, College of Medicine, University of Nigeria, Enugu Campus, Enugu, Nigeria; 8 Fakultat III, Hochschule Hannover - University of Applied Sciences and Arts, Hannover, Germany

**Keywords:** HIV, Antiretroviral therapy, Cell phone, Text-message, Medication adherence, Physical exercise adherence, Quality of life

## Abstract

**Background:**

Mobile text reminder (SMS) system is considered a viable strategy for targeting/facilitating healthy behavioural change including adherence to prescribed physical exercises (PE) and medication (antiretroviral therapy-ART) which should improve the quality of life (Qol) in people living with HIV/AIDS(PLWHA). Thus, the literature was appraised for evidence of SMS effectiveness in improving ART and PE adherence behaviours and QoL in PLWHA.

**Methods:**

Eight databases–AMED, CINAHL, Cochrane Library, EMBASE, EMCARE, Ovid MEDLINE, PsycINFO, and PubMed-were searched up to December 2020, using the Preferred Reporting Items for Systematic Review and Meta-Analysis (PRISMA) protocol.This review included only randomised control trials (RCTs) investigating the effectiveness of SMS in improving QoL or PE or ART adherence behaviour or a combination of these variables in PLWHA >18 years. Two independent reviewers determined the eligibility of the studies. Data were extracted and the quality of the study was assessed with the Physiotherapy Evidence Database (PEDro) tool. The primary outcomes were ART and PE adherence behaviours while the secondary outcome was QoL.

**Result:**

A pooled estimate of effect was not calculated due to the heterogeneity of methods and outcome measures. Therefore, a narrative synthesis of ten studies that met the inclusion criteria (n = 1621 participants at study completion) comprising males/females, aged ≥ 18 years, was done. There was a significant improvement in ART adherence behaviour except in three underpowered studies. Only the SMS interventions that were developed using the Starks 3-steps Adherence model was associated with positive outcome. The only study that evaluated QoL was underpowered and reported no significant change while there were no RCTs on PE.

**Conclusion:**

Effects of SMS intervention trends towards a significant improvement in ART adherence behaviour in PLWHA. It is plausible that SMS reminders developed using the broader framework of the interpersonal health behaviour theory(ies) may have positive outcome. Nevertheless, the observed heterogeneity in the methods/outcome measures warrants a cautious interpretation of the findings. There is a lack/paucity of RCTs and therefore no evidence in support of the effectiveness of SMS intervention in improving PE adherence and QoL. *Registration number* NPLASY202060016.

**Supplementary Information:**

The online version contains supplementary material available at 10.1186/s12879-021-06563-0.

## Background

In managing chronic diseases, behavioural adaptation to ensure adherence to prescribed intervention is often the key link to success or failure of treatment. In Human immune deficiency virus/Acquired immune deficiency syndrome (HIV/AIDS), the two mainly prescribed treatment is highly active antiretroviral therapy (HAART) and lifestyle modifications that promote physical activity [1–3]. HAART slows down disease progression, prevents HIV transmission and boosts immunity [4]. Similarly, physical activity/exercises have been found to improve bone health and immune function [5], mood [6], body composition [7], function [8] and self-rated quality of life [9]. Therefore, adherence to these interventions is an important index for good treatment outcomes and possibly the patients’ survival. However, as with other chronic conditions, adherence to remedial interventions has been the major problem of patients and holds the key to effective clinical management of HIV-infected individuals. The mobile text reminder (SMS) system has been recommended as an intervention strategy that will ensure a behavioural change that promotes adherence to treatment prescriptions. Several authors [10] expressed the view that high growth in mobile technology has provided another tool for chronic disease management, including enhancing adherence to treatment regimens especially in resource-limited settings [4] and therefore, the literature was appraised to guide practice.

SMS was considered important because targeting an individual with repetitive information overtime has been found to influence the action of the memory neural circuits which may be required for a behaviour change towards the desired adherence habit [11–13]. Therefore, behavioural change models/theories should be employed to identify and explain the changes arising from using the SMS and identify its influences on the outcomes and select the population likely to benefit from it. A major behavioural change model/theory that informs SMS reminders for treatment adherence is the health belief model [14]. This model's key elements include perceived susceptibility, perceived severity, perceived benefits, perceived barriers, cues to action, and self-efficacy. These elements help identify key decision-making points that influence health-seeking behaviour [14], some of which are targeted while using mobile text reminders to enhance treatment adherence. The expectation that a person will take a health-related action to address the individual’s perceptions of the threat posed by HIV-related disability, or to realise the benefits of taking preventive action by participating in physical activity or compliance to medication, is calibrated with cultural, socioeconomic, and environmental factors which form and modify the decision to act.

There is a lack of synthesised evidence from the literature on the effectiveness of mobile text reminders as an adherence strategy for medication and physical exercise prescriptions in HIV conditions. Therefore, it is unknown whether this strategy is effective as a behavioural change intervention that could be explored to the advantage of PLWHA, hence this study. The current review sought to address the following main review question: Is mobile text reminder effective in improving quality of life (QoL), adherence to HAART medication and physical exercise prescription in People living with HIV/AIDS (PLWHA) based on reports from studies published in the databases from 1990 to August 2019? To answer the review question, specific review objectives included determining the effects of SMS,—compared to usual care, on adherence to HAART medication and physical exercise, and quality of life in PLWHA.

## Methods

This systematic review was certified according to the international Platform of Registered Systematic Review and Meta-analysis Protocols (INPLASY register) (registration number: INPLASY202040048). INPLASY202060016.

### Eligibility criteria

Eligibility criteria considered for selecting studies in the review include:

### Inclusion criteria

i. Type of study:
  Original research manuscripts in peer-review journals and conferences proceeding were included if published in the English Language. This review included studies whose research design was a randomised control trial (RCT). Studies for the review of the following objectives were evaluated: the effect of mobile text reminders on—(1) quality of life, (2) adherence to ART medication, and (3) adherence to physical exercise prescription in PLWHA.
ii. Participants:
  Studies involving human participants aged ≥ 18 years were included in this review. Only studies that investigated PLWHA who have initiated ART were considered. There were no specific limitations concerning the setting of the studies to be investigated. Thus, studies carried out in clinics, health centres, hospitals, or community settings were also included.
iii. Intervention:
  RCTs that accessed or evaluated the impact of mobile text reminders in HIV patients were included. Inclusion was not restricted to a type, frequency, and duration of intervention or follow-up period after the intervention.
iv. Comparator:
  Studies comparing mobile text reminders with other treatment options, including usual care or no treatment were included in this systematic review.
v. Duration of intervention:
  There was no confinement on the length of the intervention’s administration, and the follow-up should be ≤ 6 months post-intervention.
vi. Outcome measures:
  The primary outcomes of interest of this review were ART and physical exercise adherence behaviours. The secondary outcome was quality of life. Thus, studies that also investigated the effects of the intervention on quality of life were included in this review. Studies were included in the review regardless of the type of the outcome measures utilised, as long as any of the outcomes of interest were accessed.

### Exclusion criteria

- Studies not published in the English language
- Studies that included in addition to mobile text reminders, other behavioural change components or technology-based reminder systems.
- Non-randomised controlled trials, pre-test post-test designs, crossover designs and other quasi-experimental studies were excluded
- In the case of similar publications from the same study, the most recent or most comprehensive publication was used.

### Information sources and search strategy

An extensive search strategy, which was formulated to identify applicable studies to be used for the review was piloted (Additional file 1: Appendix I) and implemented. The search strategy formulated to search the bibliographic database and grey literature was conducted utilising keywords, terms from medical subject heading (MeSH), with a combination of Boolean logic in the title, abstract and text for the population, intervention, study design, and outcomes. This strategy was used differently for the three selected outcomes. PubMed search strategy is shown in Additional file 1: Appendix I. This strategy was modified to conform to the syntax and subject heading of additional databases. Eight bibliographic databases were searched up to December 2020, using the Preferred Reporting Items for Systematic Review and Meta-Analysis (PRISMA) protocol. The searched databases included: Cochrane Library, EMCARE, PsycINFO, Ovid MEDLINE, Allied and Complementary Medicine Database (AMED), Cumulative Index to Nursing and Allied Health Literature (CINAHL), EMBASE, and PubMed, trial registers and directory of open-access repository websites. The search was implemented by two reviewers—NSC and EFE—employing controlled vocabularies and keywords: Seropositive, HIV/AIDS, exercise intervention, exercise program, resistance exercises, strengthening exercises, physical exercises, aerobic exercises, mobile text reminders, SMS, and QoL. Additionally, snowball searches were executed from the bibliographic references of the identified studies and grey literature. The selection of studies for inclusion in this review was based on eligibility criteria. This procedure was implemented following the guidelines in the Cochrane Handbook rules for Systematic Reviews [15] and advice for Health Care Review by the Centre for Reviews and Dissemination [16].

### Study record, selection process, and data management

**Fig. 1:**
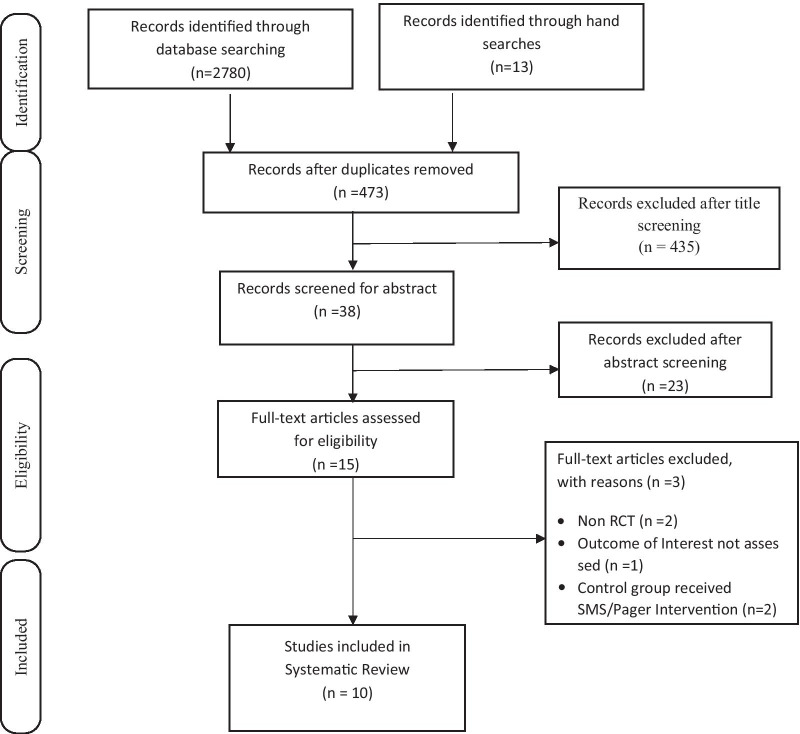
PRISMA Flow Chart for medication adherence, physical exercise adherence, quality of life

Literature search results were exported into RefWorks™ to check for duplication of studies. Considering the inclusion criteria, eligibility questions, and structures for the studies, considerations to the two levels of eligibility assessment were produced, piloted, and if required, refined. Bibliographic records were transferred from RefWorks™ into Microsoft Excel® to simplify the data organization. The selected papers were screened in two steps. The first step involved the screening of titles and abstracts of the selected papers based on the inclusion and exclusion criteria. The potentially important articles were identified by E.F.E (reviewer 1). The results of the first screening were independently cross-checked by I.F.O (reviewer 2) based on the review eligibility criteria. The second step of the screening process involved a full-text screening of the articles identified in the first step by reviewer 1. Also, reviewer 2 independently conducted a full-text screening of the identified articles to verify the findings of reviewer 1. Differences of opinions occurring at any stage regarding inclusion or exclusion were resolved by discussion and reflection, in consultation with SCI (reviewer 3). The reasons for excluding studies were properly documented in the PRISMA flow chart (Fig. 1).

### Quality appraisal for included studies

The methodological thoroughness or rigour of the included studies in this review was judged using the Physiotherapy Evidence Database (PEDro) quality appraisal tool. There are 11 items on the PEDro scale which measure the external and internal validity of any clinical trial. The first item on the PEDro evaluates external validity while the ten remaining items measure the internal validity. Apart from the first item, every satisfying criterion on the Pedro scale is given one point adding up to a maximum score of 10. The higher the score, the better the quality of the study and the following point scale was used: 6–10 (High); 4–5 (fair or moderate); ≤ 3 (poor). A point for a particular criterion was awarded only if the article explicitly reported that the criterion was met. One point was given for each “yes” answer and zero for “no”, “unclear”, and “not applicable” (N/A) answers. The final score was reported as a sum of all “yes” answers out of 10 depending on the applicable answers for each study. Scores of individual items from the critical appraisal tool were added to present the total score. The reviewers thoroughly appraised the selected studies independent of each other. Areas of differences in opinion between reviewer 1 and reviewer 2 were resolved by discussion and reflection, or by consulting with the third reviewer who swayed the decision either way.

### Data item

Data were collected from variables including authors’ references, participants’ characteristics, inclusion and exclusion criteria, study sample size, components of the intervention, the intervention setting, who delivered the intervention, the duration of the intervention and follow-up (where available), attrition rate, aspects of outcome assessed, the outcome measurement, methods/techniques, results, conclusions and funding sources.

The variables for which data were collected include:

i. Authors
ii. Participants’ characteristics (including age range, gender, sample size)
iii. Study sample size (also groups sample size where available)
iv. Intervention (setting, blinding, intervention delivery, type of intervention, duration of intervention and components of intervention)
v. Attrition rate
vi. Control
vii. Outcome(s) assessed and outcome measures/techniques
viii. Summary of Results.
ix. Conclusions and funding sources

### Quality appraisal and risk of bias

Adopting the Cochrane Collaboration Tool for Risk of Bias Assessment (Table 8.5a of the Cochrane Handbook for Systematic Reviews of Interventions), the two reviewers evaluated the risk of bias for each of the included studies in six key domains: (1) selection bias (random sequence generation, allocation concealment); (2) performance bias (blinding of personnel and participants); (3) detection bias (blinding of outcome assessments); (4) bias due to attrition (incomplete outcome data, including dropouts and withdrawals); (5) reporting bias (selective reporting) and (6) other bias (other sources of bias not elsewhere addressed) [15]. To facilitate the assessment of the possible risk of bias for each intervention study, reviewer 1 and reviewer 2 independently collected information using the PEDro tool for assessing the risk of bias comprising sequence generation, allocation concealment, blinding, adequate follow-up, between-group comparison and selective outcome reporting [15]. The procedures undertaken to assess each domain for each study was explicitly described and rated as 'high risk' or 'low risk'. The risk of bias in a study was reported as “unclear” if there were insufficient details in the original study. In such instances, the study investigators were contacted to provide the required details. The reviewer 1 and reviewer 2 made independent judgments for the risk of bias using the stipulated criteria [15]. Areas of disagreements were resolved by discussion and reflection, or in consultation with SCI (reviewer 3).

### Data synthesis and assessment of heterogeneity

The review question on the effectiveness of mobile text reminders in improving HAART medication adherence, physical exercise adherence and quality of life in PLWHA was answered. In doing this, all quantitative study outcomes which analysed the effects of this intervention were presented, examined, and combined in a proof table. Meta-analysis was not done to find the pooled effect size across studies, using a random-effect model due to heterogeneity of methods and outcome measures. Therefore, a narrative synthesis was done.

### Data and Sensitivity analysis

A narrative synthesis was conducted in this review to interpret and explore the relationships between the findings of the heterogeneous studies following the guideline of the Centre for Reviews and Dissemination [16].

### Rating quality of evidence and strength of recommendation

The quality of evidence of the studies was not evaluated to determine the strength of recommendation in the systematic review, due to the heterogeneity of methods and outcome measures. The individual study was graded as high risk of bias or low risk of bias, and then again individual evidence statement for this review was graded from ‘High Quality’ to ‘Very Low Quality’ according to the criteria set by the PEDro tool.

### How this review is reported

This systematic review was reported using the PRISMA statement guidelines [17], with all items relevant to the review included.

Results

### Search result

The initial search yielded 2780 potential papers. Following deduplication, 473 potential papers were identified and screened for the title and abstract content. Thereafter, 458 papers were excluded, and 15 papers were found eligible. Also, 13 eligible papers were identified from further hand searches. Thus, 28 papers were read and screened for eligibility, with 10 papers meeting the review’s eligibility criteria and were included in the review (Fig. 1—PRISMA flow diagram). Reasons for exclusion of studies following full-text screening include non-randomised controlled trials (n = 2), the outcome of interest not assessed (n = 1), and the control group received SMS/Pager Intervention (n = 2).

### Characteristics of included studies

All the included studies were randomised controlled trials and contributed a total of 2190 participants comprising 919 males and 1260 females, while the gender of 11 participants was undisclosed (Table 1). The number of participants in the 10 included studies ranged from 29 [18] to 538 [19] PLWHA. Participants were ≥ 18 years of age, with most of them on ART for at least 1 month. The clinical characteristics between the intervention and control groups do not differ significantly at baseline.

**Table 1:** Study characteristics

Author (year)	Location of study	Participants Number (n) Gender Age in years/mean age ± SD Period on ART Attrition rate	Intervention Number allocated Lost to follow-up (died)	Period of intervention	Outcome measure	Outcome tool	Theories and models of behaviour change used or adopted	Conclusion
Gross et al. (2019). [23]	Multinational: Brazil Haiti India Kenya Malawi South Africa Thailand Uganda Zimbabwe	N = 521 Male (272); female (249) Age: ≥ 18 years Has previously taken and had resistance to NRTI or NNRTI, who were currently accessing a second-line protease inhibitor-containing Regimen, which they had been on for at least 24 weeks with no previous darunavir or etravirine exposure Attrition rate = NR	G_1_: mobile text reminders (SMS reminder and flashback system once daily for 8 weeks, thrice weekly for another 8 weeks and then once weekly till the 48th week) Number allocated to g_1_: 257 The number lost to follow-up: 8 (11 died) G_0_: standard of care adherence support Number allocated to g_0_: 264 The number lost to follow-up: 12 (12 died)	48 weeks	Medication adherence	Self-report adherence questionnaire	NR	Two-way adherence intervention did not show any clinically relevant benefit
Ruan et al. (2017) [20]	China	N = 100 Male (59); female (41) Age: ≥ 18 years Has been on ART for not more than 3 months Attrition rate = NR	G_1_: mobile text reminder (SMS for 6 months + usual care) Number allocated to g_1_: 50 The number lost to follow-up: 3 (2 death and 1 withdrawal) G_0_: usual care which includes: regular health education in the clinic including informational pamphlets, psychological support and personalized health education from nurses and physicians) Number allocated to g_0_: 50 The number lost to follow-up: 3 (1 withdrawal)	6 months	Medication adherence	CPCRA adherence self-report questionnaire VAS on a 100- point scale	Starks et al.’s 3-steps adherence model (centres on 3 steps: step 1- knowledge, step 2- motivation, step 3-proximal cue to action = medication adherence). The SMS intervention was developed based on this model	SMS showed significant efficacy in improving adherence to ART medication in people living with HIV
Nsagha et al. (2016) [26]	Cameroon	N = 90 Male (35); female (55) Age: ≥ 18 years Has been on for at least 1 month Attrition rate = NR	G_1_: mobile text reminder (standard treatment and care + 4 times weekly educative SMS for 4 weeks) Number allocated to g_1_: 45 The number lost to follow-up: NR G_0_: standard treatment and care Number allocated to g_0_: 45 The number lost to follow-up: NR	1 month	Medication adherence	Self- reported using an interviewer-administered questionnaire	NR	SMS significantly improved adherence to an antiretroviral, a key constraint that affects adherence to antiretroviral medication
Sabin et al. (2015) [27]	China	N = 119 Male (76); female (43) Age: ≥ 18 years Has been receiving or initiating ART for the first time Attrition rate = NR	G_1_: (comprised of suboptimal adherence group < 90% medication adherence and optimal adherence group > 90% medication adherence) received, real-time SMS reminder triggered by a 30 min delay in medication + adherence counselling Number allocated to g_1_: 63 The number lost to follow-up: 1 G_0_: (comprised of suboptimal adherence group < 90% medication adherence and optimal adherence group > 90% medication adherence) received, usual care + adherence counselling Number allocated to g_0_: 56 The number lost to follow-up: 2	6 months	Medication adherence	Wise-pill device	NR	The use of triggered SMS reminders significantly improved antiretroviral therapy adherence in the HIV population
Moore et al. (2014) [24]	USA	N = 58 Male (44); female (6) The gender of 8 participants who were not included in the analysis was not reported Age: ≥ 18 Has been on ART, period not specified Attrition rate = NR	G_1_: psychoeducation + daily text message medication reminder and mood inquiries for 30 days Number allocated to g_1_: 30 The number lost to follow-up: 0 G_0_: standard of care adherence psychoeducation and daily text mood inquiries Number allocated to g_0_: 28 The number lost to follow-up: 2	30 days	Medication adherence	MEMS Self-reported adherence using VAS	Theory of planned behaviour which posits That behaviour is driven by behavioural intentions and that Individual motivational factors interact with cognitive Impairment, mood disruption, and substance use to create Both intentional and unintentional nonadherence. The SMS intervention was constructed based on this theory	Daily contact via text messaging is feasible even in difficult populations. Text messaging in conjunction with psychoeducation improves ART doses timing in a group of individuals who are at high risk for nonadherence to important medications (ART) Both groups showed high levels of overall 30-day MEMS adherence but did not significantly differ for ARV medication adherence
Maduka and Tobin-West., (2013) [22]	Nigeria	N = 104 Male (45); female (59) Age: ≥ 20 Has been on HAART for at least 3 months before enrollment Attrition rate = NR	G_1_: adherence counselling (one-on-one monthly for 4 months) and text message reminder (twice weekly for 4 months) via an internet-based bulk SMS facility—‘light edge SMS’ powered by light edge systems Number allocated to g_1_: 52 The number lost to follow-up: 2 G_0_: standard care Number allocated to g_0_: 52 The number lost to follow-up: 8	4 months	Medication adherence	Self-reported adherence questionnaire	NR	A combination of counselling and text message reminders significantly improved drug adherence among non-adherent HIV patients on HAART
Mbuagbaw et al. (2012) [21]	Cameroon	N = 200 Male (53); female (147) Age: ≥ 21 years Has been on ART for at least 1 month Attrition rate = NR	G_1_: mobile text reminder (motivational SMS; weekly for 6 months + usual care) Number allocated to g_1_: 101 The number lost to follow-up before phone prompt: 59 The number lost to follow-up after phone prompt: 21 G_0_: usual care (regular ART counselling and home visits) Number allocated to g_0_: 99 The number lost to follow-up before phone prompt:57 The number lost to follow-up after phone prompt: 21	6 months	Medication adherence Quality of life	VAS Self-report Pharmacy refill data SF-12 QOL assessment form	Health belief model. The SMS intervention was designed based on a focus group discussion and this model	There was no significant improvement in the quality of life nor adherence to ART after 6 months of SMS intervention However, sensitivity analysis showed a slight improvement in medication adherence in the intervention group compared to the control group
da costa et al. (2012) [18]	Brazil	N = 29 Male (0); female (29) Age: 34.62 ± 6.92 Patients on first or second ART regimen Attrition rate = NR	G_1_: mobile text reminder (SMS messages; 5 times a week for 4 months) Number allocated to g_1_: 14 The number lost to follow-up: 1 (5 participants did not receive allocated intervention) G_0_: no SMS intervention Number allocated to g_0_: 15 The number lost to follow-up: 2	4 months	Medication adherence	Self-reported adherence questionnaire Pill counting MEM	NR	No significant change in the three outcome measures for medication adherence was reported However, the intervention stimulated more participants in the intervention group to be adherent to their treatment for at least 4 months of the study period
Pop-eleches et al. (2011) [25]	Kenya	N = 431 Male (148); female (280) The gender of 3 participants who were not included in the analysis due to faulty MEMS were not reported Age: > 18 years Initiated ART for less than 3 months before enrollment Attrition rate = NR	G_1_: mobile text reminder (SMS; either short or long messages at daily or weekly frequency for 48 weeks) The number of patients excluded due to faulty MEMS: 3 Number allocated to short daily messages: 70 The number lost to follow-up: 18.6% Number allocated to long daily messages: 72 The number lost to follow-up: 16.7% Number allocated to short weekly messages: 72 The number lost to follow-up: 22% Number allocated to long weekly messages:74 The number lost to follow-up: 10.8% G_0_: no intervention Number allocated to g0: 139 The number lost to follow-up: 14.4%	48 weeks	Medication adherence	Medication event monitoring system (MEMS)	NR	There was a significant change due to the intervention on the participants who received weekly SMS reminders but no significant change was found in those participants who received daily SMS reminders nor the control group
Lester et al. (2010) [19]	Kenya	N = 538 Male (187); female (351) Age: > 18 years Initiating ART for the first time Attrition rate = 21%	G_1_: mobile text reminder (SMS support service; weekly for 12 months) Number allocated to g_1_: 273 The number lost to follow-up: 17 G_0_: standard care Number allocated to g_0_: 265 The number lost to follow-up: 27	12 months	Medication adherence	Self-reported adherence questionnaire	NR	SMS intervention in PLWH significantly improved adherence to ART compared to patients who received the standard care alone

*AR* attrition rate, *ART* antiretroviral therapy, *CPCRA* Community Programs for Clinical Research on Aids, *G*_*1*_ intervention group, *G*_*0*_ control group, *MEMS* medication event monitoring system, *MEM* micro-electronic monitors, *NR* not reported, *NRTI* nucleoside analogue reverse transcriptase inhibitors, *NNRTI* non-nucleoside reverse transcriptase inhibitors, *SMS* short message services, *SD* standard deviation, *SF-12 QOL* Short Form-12 Quality of Life Assessment Form, *VAS* visual analogue scale

### Outcome measures

Different studies utilised different outcome measurement tools for evaluating the same outcome. For instance, ART adherence behaviour was assessed using: self-report [18–24]; medication event monitoring system [24, 25]; Visual analogue scale [20, 21, 24]; pill count [18]; micro-electronic monitoring [18]; pharmacy refill data [21]; community programs for clinical research on AIDS adherence self-report questionnaire [20]; quality of life [21]; interviewer-administered self-report questionnaire [26] and wise-pill monitor [27].

### Quality appraisal and risk of bias assessment

The PEDro tool for the risk of bias assessment was adopted for this review (Table 2). Three studies [20, 25, 26] were judged as moderate quality studies while the other seven studies [18, 19, 21, 22, 27] were judged as high-quality studies.

**Table 2:** Quality Appraisal/Risks of Bias of included studies (PEDro Tool)

Study	Eligibility Criteria	Random allocation	Concealed allocation	Baseline comparability	Blinding of subjects	Blinding of therapist	Blinding of assessors	Adequate follow-up	Intention to treat analysis	Between-group comparison	Point estimates and variability	Total score	Quality index
Gross et al. (2019) [23]	Yes	Yes	No	Yes	No	Yes	No	Yes	Yes	Yes	Yes	7/10	High
Ruan et al. (2017) [20]	Yes	Yes	No	Yes	No	No	No	No	Yes	Yes	Yes	5/10	Moderate
Nsagha et al. (2016) [26]	Yes	Yes	No	Yes	No	No	No	Yes	No	Yes	No	4/10	Moderate
Sabin et al. (2015) [27]	Yes	Yes	Yes	Yes	No	No	No	No	Yes	Yes	Yes	6/10	High
Moore et al. (2015) [24]	Yes	Yes	No	Yes	No	No	Yes	Yes	No	Yes	Yes	6/10	High
Maduka and Tobin-West., (2013) [22]	Yes	Yes	Yes	Yes	No	Yes	Yes	Yes	Yes	Yes	Yes	9/10	High
Mbuagwu et al. (2012) [21]	Yes	Yes	Yes	Yes	No	Yes	Yes	Yes	Yes	Yes	Yes	9/10	High
da Costa et al. (2012) [18]	Yes	Yes	No	Yes	No	Yes	Yes	No	No	Yes	Yes	6/10	High
Pop-Eleches et al. (2011) [25]	Yes	Yes	No	Yes	No	No	No	Yes	Yes	Yes	No	5/10	Moderate
Lester et al. (2010) [19]	Yes	Yes	Yes	Yes	No	No	Yes	Yes	Yes	Yes	Yes	8/10	High

The PEDro scale was used to determine and summarize the quality of the included studies

NB: Eligibility criteria is not awarded a score in the scoring

### Eligibility criteria

Authors of the 10 studies [18–27] reported on the inclusion and exclusion criteria used in recruiting and screening participants for their respective studies. Hence, a low risk of bias was evident for all included studies.

### Random allocation

All included studies [18–27] reported using the randomisation process to allocate the eligible participants into the different groups (Experimental and control groups). Thus, low risk for selection bias was also evident for the included studies.

### Concealment of allocations

Only four studies [18, 19, 21, 22] reported concealment from the participants in the different groups they were randomly allocated to. This may affect the overall selection bias since the greater number (six) of the included studies did not report on concealment allocation.

### Baseline similarity

All participants in the included studies [18–27] have similar baseline characteristics of the measured outcome variables. Thus, the groups were relatively equivalent at baseline.

### Bias on blinding

Four studies [18, 21–23] reported on the blinding of therapists during the intervention period, but none reported on blinding the  participants. Five studies reported on blinding the assessors [18, 19, 21, 22, 24] and thus have a low risk of bias.

### Between-group comparison

All included studies [18–27] reported on performing the between-group comparison.

### Adequate follow-up

Seven of the ten included studies [19, 21–26] reported having an adequate follow-up.

### Intention to treat analysis

Seven of the ten included studies [19–23, 25, 27] indicated that they did not conduct the intention to treat analysis.

### Point estimate and variability

Two of the included studies [25, 26] reported the desired outcomes without using point estimate and variability.

### Outcomes reported in included studies

All included studies [18–27] reported on ART adherence behaviour while none reported on physical activity adherence (Table 3). One study reported on the quality of life [21].

**Table 3:** Data extraction of findings (except where specified, results are presented as Int. group vs. Cont. group)

Study	Timepoint	Medication adherence and outcome	Physical activity adherence and outcome	Quality of life and outcome
Gross et al. (2019) [23]	Immediately post-intervention at week 48	100%: Using self-reported adherence n (%): [Int. 174(73) vs. Cont. 173(69)]; p = 0·89		
Ruan et al. (2017) [20]	Immediately post-intervention at month 6	100%: Using CPCRA adherence n (%): [Int. 42(89.3) vs. Cont. 34(72.3)]; Z = 2.208; p = 0.027 80%-99% Using CPCRA adherence n (%): [Int. 3(6.4) vs. Cont. 3(6.4)]; Z = 2.208; p = 0.027 < 79% Using CPCRA adherence n (%): [Int. 2(4.3) vs. Cont. 10(21.3)]; Z = 2.208; p = 0.027 Using Visual Analogue Scale (VAS) mean ± SD: [Int. (98.72 ± 2.35) vs. Cont. (93.11 ± 6.51)]; Z = 2.735; p = 0.006		
Nsagha et al. (2016) [26]	Immediately post-intervention at week 4	Using the interviewer-administered self-report questionnaire %: [Int. 64.4% vs. Cont. 44.2%] Z = NR; p = 0.05		
Sabin et al. (2015) [27]	Immediately post-intervention at month 9 following 3 months of pre-intervention	Using wise-pill device mean ± SD: [Int. (96.2 ± 6.4) vs. Cont. (89.1 ± 15.9)]; Z = NR; p = 0.003		
Moore et al. (2015) [24]	Immediately post-intervention at day 30	Using MEMS mean ± SD:: [Int. (86.2 ± 12.7) vs. Cont. (84.8 ± 18.1)]; cliff’s d = 0.01; p = 0.95 Using self-reported VAS mean ± SD: [Int. (95.8 ± 6.6) vs. Cont. (92.4 ± 13.0)]; Z = NR; p = 0.44		
Maduka and Tobin-West., (2013) [22]	Immediately post-intervention at month 4	Using self-reported adherence questionnaire n (%): [Int. 40(76.9) vs. Cont. 29(55.8)]; Z = 0.224; p = 0.022		
Mbuagbaw et al. (2012) [21]	Immediately post-intervention at month 6	Using Visual Analogue Scale (VAS) n (%): [Int. 72(71.3) vs. Cont. 66(66.7)]; Z = NR; p = 0.542 Using self-report n (%): [Int. 80(79.2) vs. Cont. 78(79.0)]; Z = NR; p = > 0.999 Using pharmacy refill data mean ± SD: [Int. (3.8 ± 1.48) vs. Cont. (3.7 ± 1.34)]; Z = NR; p = 0.617		Using SF-12 scale score Quality of life assessment form: [Int. (3.79 ± 0.585) vs. Cont. (3.75 ± 0.583)]; Z = NR; p = 0.629
da Costa et al. (2012) [18]	Immediately post-intervention at month 4	Using self-reported adherence n (%): [Int. 8(100.00) vs. Cont. 12(92.31)]; Z = 0.8038; p = 0.4215 Using pill counting n (%): [Int. 5(62.50) vs. Cont. 6(46.15)]; Z = 0.7284; p = 0.4664 Using micro-electronic monitoring systems (MEMS) n (%): [Int. 6(75.00) vs. Cont. 7(53.85)]; Z = 0.9694; p = 0.3324		
Pop-eleches et al. (2011) [25]	Immediately post-intervention at week 48	Using the medication event monitoring system (MEMS) n/N: 4 intervention groups (daily short messages, weekly short messages, daily long messages and weekly long messages) with the control group Summary group (All daily reminder groups, irrespective of message length): [Int. 0.41 vs. Cont. 0.40]; Z = NR; p = 0.92 Summary group (All weekly reminder groups, irrespective of message length): [Int. 0.53 vs. Cont. 0.40]; Z = NR; p = 0.03 Summary group (All short reminder groups, irrespective of message frequency): [Int. 0.47 vs. Cont. 0.40]; Z = NR; p = 0.27 Summary group (All long reminder groups, irrespective of message frequency): [Int. 0.47 vs. Cont. 0.40]; Z = NR; p = 0.24 Subgroups Subgroup 1 (daily short messages): [Int. 0.40 vs. Cont. 0.40]; Z = NR; p = 0.97 Subgroup2 (weekly short messages): [Int. 0.53 vs. Cont. 0.40]; Z = NR; p = 0.07 Subgroup3 (daily long messages): [Int. 0.42 vs. Cont. 0.40]; Z = NR; p = 0.85 Subroup4 (weekly long messages): [Int. 0.53 vs. Cont. 0.40]; Z = NR; p = 0.08		
Lester et al. (2010) [19]	Immediately post-intervention	Using self-reported questionnaire n (%): [Int. 168(62) vs. Cont. 132(50)]; Z = NR; p = 0.006		

*Int.* intervention group, *Cont.* control group, *p* p value, *n* number of participants, *N* total number of participants, *Subgroup1* daily, short SMS, *Subgroup2* weekly, short SMS, *Subgroup3* daily, long SMS, *Subgroup4* weekly, long SMS, *SMS* short message service, *NS* not significant, *NR* not reported, *CPCRA* community programs for clinical research on AIDS adherence self-report questionnaire

### Effects of intervention

Except where otherwise stated, the effects of the intervention are reported as a comparison of the intervention group versus the control group (Table 3).

I. Medication adherence
  Ten studies [18–27] provided data on ART adherence after SMS reminders. Five [19, 20, 22, 25–27] of the ten included studies reported that SMS reminder systems were associated with significantly improved ART adherence behaviour in PLWHA. One high-quality trial [22] reported a significant change or improved adherence using self-reported adherence questionnaire (χ^2^=5.211, p = 0.022). Similarly, two studies [19, 20] reported that mobile text reminder had a significant effect on adherence to medication, provided the participants are adequately followed up. One of them is a moderate-quality trial [20] which used the community programmes for clinical research on AIDS adherence self-report questionnaire: p = 0.027; and visual analogue scale: p = 0.006, as outcome measures. The other study is a high-quality trial [19] which used self-report (p = 0.006), as the outcome measure. Another moderate-quality trial [26] reported a significant change in ART adherence behaviour using an interviewer-administered self-report questionnaire (p = 0.05) as the outcome measure. One high-quality trial [27] reported a significant change in ART adherence behaviour using a wise-pill monitor: (group 1, p = 0.039; group 2, p = 0.028) as the outcome measure.
  Some studies reported participants’ preference for privacy and requested certain codes to be used for some words. However, one moderate quality study [25] reported a mixed result. It reported a significant improvement in ART adherence behaviour in one intervention group (weekly SMS: p = 0.03) out of the four intervention groups, using the medication event monitoring system as the outcome measure. The remaining three intervention groups (Daily SMS: p = 0.92, Short SMS: p = 0.27, Long SMS: p = 0.24) showed no significant change.
  Four studies [18, 21, 23, 24] reported that ART adherence behaviour was not significantly improved by SMS reminders. One high quality trial [21] reported no statistically significant change using different outcome measures which included: visual analogue scale: p = 0.542; self-report: p = 0.999; and pharmacy refill data: p = 0.617. Nevertheless, a sensitivity analysis was conducted which revealed that more participants in the SMS group attained adherence of >90% at 6 months. Another high-quality trial [18] reported no significant change using different outcome measures including: self-report: p=0.2435, pill counting: p=0.6038 and micro-electronic monitoring: p = 0.1946. A high-quality trial [23] reported no significant change (p = 0·89) in ART adherence behaviour using a self-report questionnaire as the outcome measure. A high-quality study [24] reported that both the SMS-supported group and control group showed high levels of overall 30-day MEMS adherence and did not significantly differ for ARV/ART adherence behaviour using MEMS (p = 0.95) and self-reported VAS (p = 0.44) as outcome measures. However, there was a significant reduction in ART dose timing windows in a group of individuals who were at high-risk for nonadherence. They found that greater dose timing windows were associated with poorer overall adherence or increased chances of missing a dose.
  Across the included studies, the following prescriptions of mobile text reminder were associated with positive outcomes for medication adherence: Mobile text—personalised SMS reminder: 3–5 times/week × 6 months, when delivered 30 mins before medication time plus usual care (regular health education in the clinic including informational pamphlets, psychological support and personalised health education from nurses and physician) [20], OR either short or long delivered SMS at daily or weekly frequency × 48 weeks [25], OR text message reminder (twice weekly for 4 months) via an internet‑based bulk SMS facility—‘Light Edge SMS’ plus adherence counselling (one-on-one monthly for 4 months) [22] OR mobile text reminder (SMS support service; weekly for 12 months) plus adherence counselling [19] OR 4 educative SMS/week × 4 weeks [26] OR real-time SMS reminder triggered by a-30 min delay in medication plus adherence counselling × 6 months [27].
  Effectiveness of theory-based SMS reminders in improving ART adherence behaviour
  Some behaviour change theories or models were used in only three [20, 21, 24] of the ten included studies to either rationalise/illuminate the basis of the behaviour change intervention or design the intervention. Generally, the included studies had a common aim of improving ART adherence behaviours but varied in their specific objectives concerning settings, outcome measures and quality. Therefore, the narrative data were synthesised to achieve our review’s specific objectives, which provided the context for reporting the results below. First, studies on SMS reminders utilizing theories or models of behaviour change to improve adherence to ART in PLWHA were charted. Subsequently, the evidence of the effectiveness of the SMS reminders in improving ART adherence behaviour was assessed, where the behaviour change theories or models were used.
II. *HIV staging and theory-based interventions*
  Only one study [20] of the three studies that utilised theory-based intervention failed to include the staging of the HIV disease among the participants. It was not indicated whether the participants were asymptomatic to give a measure of the disease severity which should have implications for the applied behaviour change model, especially a motivation or demotivation to act. Two studies provided information on the disease characteristics including the number of participants with AIDS [24] or AIDS-defining illness [21].
III. *The theoretical basis of the SMS reminders:*
  The SMS reminders in all three studies [20, 21, 24] were informed by the health-related behaviour change theories or models. Two studies [20, 24], provided comprehensive information on how they utilised the health-related behaviour change theories or models in developing SMS reminders. All three studies [20, 21, 24] reported that the SMS reminders were informed by only a single theory or model. One study [20], reported that the SMS reminders were developed using Stark’s 3-steps Adherence model [28]. The authors [20] indicated that they addressed the three components of the model via interactive and comprehensive SMS reminders. Another study [24], indicated that the SMS reminders were informed by individual motivational factors as derived from the Theory of Planned Behavior. One study [21] specified that the SMS reminders were informed by the Health Belief Model, taking into account the perceived beliefs of the target participants for each intervention.
IV. *Theory and the SMS reminders design*
  All three studies [20, 21, 24] indicated that a single theory or model was used to design SMS reminders. They all utilized the behaviour change theories or models in the intervention design.
V. *Theory and the intervention or programme:*
  All three studies [20, 21, 24] used behaviour change theories or models in choosing the intervention utilised. The underpinning theoretical frameworks were those that model individuals’ health-related behaviour. The intervention/programme in one study [21] was informed by the Health Belief Model. One study [21] did not provide much detail but merely stated that a theory or model informed the intervention. Two studies [20, 24] provided comprehensive information on how the behavioural change model or theory was applied in developing the intervention design. The SMS reminder programme in one study [24] explored the individual motivational factors using the Theory of Planned Behavior. The other study [20] was based on Starks et al.’s 3-steps Adherence model.
VI. *Applicability of the health-related theory in the evaluation of the outcomes*
  One study [20] provided information on how the outcomes of the study were evaluated using the health-related behaviour change theory or model—Starks et al.’s 3-steps Adherence model. In another study [24], the specific component of the health-related behaviour change theory or model—Theory of Planned Behavior, was not primarily evaluated and many of these components were watered down. One study [21] merely stated that the health belief model was used, but did not evaluate the outcome using the model.
VII. *Targeted Populations or sub-populations*
  The interventions in all three studies targeted a wide range of populations. Over one-fifth of the interventions targeted more than one population (HIV infected population and also had a history of lifetime substance abuse/dependence). The end-users were the population targeted by all of the interventions. Population types ranged from adolescents to older adults. None of the studies neither segmented their target populations into sub-populations nor delivered separate interventions to a given group of participants.
VIII. *Categories of behaviour targeted by the SMS reminders*
  ART medication uptake was the most frequently targeted behaviour for change in all three studies [20, 21, 24]. Attempts to alter behaviour, favouring ART medication, psychotropic (PSY) adherence and dose timing was the focus of one study [24]. One study [20] specifically targeted the adoption of disease mitigation behaviour and increasing awareness and knowledge about the disease as a way of improving ART medication adherence. The same study [20] also targeted participants’ actual ingestion of the correct medication dose at the right time, through interactive text message with the care provider, as a way of increasing motivation for medication adherence. Only one of the reviewed studies [24] targeted more than one behaviour for change—including ARV/ART adherence behaviour and psychotropic adherence. It was also targeted at improving other disease outcomes such as the CD_4_^+^ cell count.
IX. *Health communication channels, activities and settings used in the studies*
  Health communication was not considered as an inclusion criterion for studies included in this review. Nevertheless, two of the studies [20, 24] described some health communication channel or activities. One study [20] used interactive text message as the communication channel between the interventionists and the participants, and which were recorded in the study log. In the other study [24], the researchers used phone calls and interactive text messages to communicate with the participants. The most common activity was face-to-face psychoeducation/health education sessions which focused on the importance of medication adherence [20, 24]. In one study, the researchers used a standard script and a PowerPoint presentation [24]. Another study [20] utilised informational pamphlets. The healthcare settings were the most common settings for interventions in the included studies (n = 3). The three intervention studies [20, 21, 24] were set in hospitals.
X. *Applicability of the theory or model in the intervention*
  None of the three studies [20, 21, 24] included in this review assessed the applicability of the theory or model they applied in their studies, respectively. All three studies [20, 21, 24] merely indicated that the interventions were developed based on a given model’s framework or its applicability, but did not further evaluate it. The authors did not provide any details on how theoretical constructs were applied to their intervention and did not indicate whether and how the evaluation tools were developed based on the behaviour change theory constructs.
XI. *Was the health behaviour change objective of the SMS reminders met*
  Two [21, 24] of the three studies (or more than half—66.67%) were graded as high quality (≥ 75% overall validity). However, only one [24] high-quality study demonstrated that the intervention was successful in significantly changing the behaviour of its participants. Changed behaviour was mainly improved adherence to ART as no study measured adherence to physical exercises. Overall, patients were targeted in their home setting in the three studies: of which one study [20] reported a significant change in behaviour while two studies [21, 24] reported no significant change in ART adherence behaviour. The evidence indicated that one individual-level behaviour theory—Stark’s et al [28] 3-steps Adherence model [20]—and the Theory of Planned Behavior [24] were associated with positive outcomes, whereas the Health Belief Model [21]—was not associated with positive outcomes.
XII. *Evidence for effective interventions and associated theories/models of behaviour change to improve ART adherence behaviours, prevent or control HIV transmission and/or progression*
  The primary aim of the two high-quality studies [21, 24] was to improve ART adherence behaviours. However, the secondary aim did not comprise either the prevention of HIV progression or prevention and control of HIV transmission. Therefore, it was difficult to determine whether the SMS reminders would be successful in not just achieving the primary aim of improving ART adherence behaviour but as a consequence, improving the clinical indices that characterise HIV progression (such as CD_4_ cell counts) and/or suppress its transmission (such as viral loads). The only study [20] that measured the CD_4_ cell count did not find any significant change but recorded a change in a positive direction. Among all three studies [20, 21, 24] whose SMS reminders were informed by behaviour change theories, no comparative evidence was presented to determine whether using the theory made the SMS reminders effective or not. However, charting and matching the studies according to their theoretical basis enabled a useful comparison of those with an effective SMS reminder (i.e., a successful outcome) to those without.
XIII. Quality of life
  One study [21] reported no significant difference in the QoL between the experimental group and control group using SF-12 Quality of Life Assessment form (p = 0.629) as the outcome measure.

## Discussion

Ten trials [18–27] determining the effectiveness of mobile text reminders in improving HAART medication adherence, physical exercise prescription adherence and QoL in PLWHA, were reviewed. The included studies had a low or moderate risk of bias and were mostly of high or moderate methodological quality. Most of the studies included in this review were conducted in the lower middle income (developing countries) than high income (developed) countries. This may have some implications as the trend of non-adherence to ART is common in the high-income countries [29] than the lower-middle-income countries. Nevertheless, non-adherence to ART has also been documented in lower-middle-income countries [30]. Therefore, the findings of this review have relevance in both contexts.

### Adherence to medication

The results from six [19, 20, 22, 25–27] of the ten studies included in this review demonstrate a potential for mobile text reminders to improve ART adherence behaviour. These studies revealed that mobile text reminders have a significant effect on ART adherence behaviour irrespective of the utilized outcome measurement tools. One moderate quality study considered the frequency of the intervention that would bring about an increase in adherence [25]. The study reported a significant effect of only weekly but not daily SMS reminders on ART adherence behaviour using a medication event monitoring system as the outcome measure. Habituation or the waning of a response to a regularly recurrent stimulus may explain these findings. Similarly, some participants might have considered daily messages as invasive and a nuisance rather than a cue to action resulting in the lack of success associated with the intervention. Also, poor retention habit or nature is likely in this population due to long-lasting or chronic management of the disease. The reasons for this are diverse and depends on the individual (e.g., pain, stigma, discrimination, mood disorders), interpersonal (e.g., quality of patient care communication or patient-provider rapport) and structural (socio-economic status, insurance entitlement) factors.

In the above context, the health belief model predicts that barriers to mobile text reminders may lie with the environmental and personal characteristics of an individual. This may improve with counselling and training sessions on the benefits of adherence to medication/physical exercise, and provides sufficient cues for a positive response when mobile text reminders are deployed in patient management. However, the above study [25], just like six others [18, 19, 22, 23, 26, 27], was not underpinned by any behaviour change model. This would have provided a guiding theoretical framework for identifying and addressing all or some of the barriers to the implementation of the SMS reminders but was not done.

Only three studies included in this review were informed by models or theories of individual-level behaviour change. There were more resemblances than contrasts concerning the variety of health behaviour change theories used between successful [20] and unsuccessful studies [21, 24]. The main differences were that one successful study [20], used the theory of interpersonal health behaviour (Starks 3-steps) in designing the SMS intervention which takes into consideration how an individual’s environment interacts with health behaviour. One study [24], using a theory of interpersonal health behaviour (Theory of Planned Behaviour), which accounts for habitual behaviours, did not report any significant difference between the intervention and control groups. However, the study [24] recorded a positive trend in ART adherence behaviour. Importantly, the study [24] found a significantly improved dose timing accuracy using SMS reminders, and further reported a direct association between dose timing window and ART adherence behaviours.

Since text messaging significantly improved dose timing accuracy, it is plausible to expect that it should likewise improve ART adherence behaviours over a longer period. More so, when it was evident that SMS reminders designed using the Theory of Planned Behavior increased responsiveness to dose timing. The projected clinical endpoints over a long time will involve better HIV disease indicators. Therefore, the short time (30 days) duration of the trials [24] could explain why this expectation was not realised, and not necessarily because the intervention was not effective. This view is strengthened by the observation that the only successful study [20] lasted (for 6 months) much longer than 30 days. However, the other unsuccessful study [21] lasted for a comparable period (i.e., 6 months) as the successful study but did not improve the ART adherence behaviour in PLWHA. The above findings suggest that when designing an SMS intervention for successful ART behaviour adherence, consideration must be given to the underlying theoretical framework and duration of implementation.

The health belief model used in one of the unsuccessful studies [21] does not consider habitual or usual behaviours that are likely to inform the decision-making process to accept a recommended action. In the context of the health belief model, the mobile text reminders can be considered as cues which influence the key decision-making points of action to achieve perceived health gains of taking preventive actions. For PLWHA, some disease preventive actions include participating in physical exercises or compliance to ART regimen. The key motive for taking either preventive or remedial actions is to address an individual’s perceptions of the threat posed by a consequent HIV-related disability. However, the health belief model does not consider behaviours that are socially acceptable and thus performed for non-health-related reasons. This may explain why the health belief model was unsuccessful when applied in developing SMS reminders for behavioural change among PLWHA.

An analysis of how the theories were used revealed that none of the studies indicated that they used a theory or model to evaluate the behaviour change interventions. Therefore, it is not known if the theories can serve or act as useful tools in providing insight grounded on the measured theoretical constructs. Two theories/models were associated with the studies [21, 24] reporting a lack of significant results. The two studies [21, 24] were graded as high quality and reported no evidence of an effect. Importantly, the two studies [21, 24] did not reveal any trends in the intervention targets to clarify the lack of success. The two studies [21, 24] used individual-level behaviour change theory—the Health Belief Model and the Theory of Planned Behavior—to inform the interventions. All the included studies demonstrated a clear focus on the PLWHA—adults (end-user) as the target for the intervention. However, there was no evidence on interventions which established and verified new theories. There was no appraising evidence on interpersonal and community-level theories and thus it was not possible to gauge their relevance in behaviour change interventions. None of the studies analysed the cost implications or cost-effectiveness of theory-based interventions, which is important considering that the majority of these studies were done in developing countries or limited-resource settings where funding is critical to the feasibility of the SMS reminders in implementing patient care.

Four high-quality studies [18, 21, 23, 24] reported no significant effect of the mobile text reminder on ART adherence behaviour, which may be related to the fact that the sample size used was powered to detect a 20% difference in adherence between the intervention and control groups but the difference found was much less. Another common similarity across the four studies [18, 21, 23, 24] is that they lasted less than a year. Thus, the duration of the trials might be insufficient to observe a significant effect, and likewise the length of time the participants have been on HAART. For instance, the multicentre/multinational (Brazil, Haiti, India, Kenya, Malawi, South Africa, Thailand, Uganda, Zimbabwe) trials [23] ended at 48 weeks. Similarly, the USA trials [24] lasted for about a month (30 days), the Brazil trials [18] ended at 4 months, while the Cameroon trials [21] ended at 6 months. However, a few of the studies reported on the effect size of SMS reminders on ART adherence behaviour when comparing the intervention group versus the control group. Although the review reports the effectiveness of mobile text reminder in improving HAART medication adherence, this should be interpreted with caution due to the following reasons:

i. Studies’ failure to report the measure of adherence for participants in both the intervention and control groups,
ii. The use of a mostly subjective measure of adherence may affect the estimate of effect because under or over-estimation can occur during the process of the interview (self-reporting) due to factors such as difficulty in remembering all the details of the drugs. This is important because participants’ self-report is the principal measure used in the studies included in this review, and various types of questionnaires were used which may elicit varied responses. Importantly, the validity of participants’ responses is questionable because they have not been compared with objective adherence measure, and patients may attempt to please their caregiver or avoid confrontation/.
iii. The use of different intervention frequencies (daily, once per week, twice per week, thrice per week) in the various studies made it difficult to compare the results across the various studies in the review. This difficulty became more obvious when it is considered that some studies reported nothing about the frequency of the prescribed intervention used.
iv. The use of different outcome measures to estimate the effect of the same intervention across the included studies in this review makes it difficult to compare effects across the groups. In recognition of this challenge, one study [19] which showed positive effects of the mobile text reminder on adherence to medication in PLWHA expressed some reservations by stating that its applicability to other countries needs to be evaluated since various outcome measures were used to assess medication adherence in contemporary literature. Therefore, there is a need for researchers to come up with a standardised outcome measure to reduce bias in the result.

Despite the above reasons, mobile text reminder has shown potential benefits in helping patients to remain adherent to treatment.

### Physical activity adherence

No study reported the effectiveness of mobile text reminders in improving physical exercise adherence in an HIV population. Therefore, it is not feasible to draw any valid scientific conclusion on this matter. However, we identified one study protocol [31] that evaluated QoL as one of the outcomes. The study protocol [31] aims to determine the efficacy of a personalized interactive mobile health intervention (iSTEP) in enhancing physical activity and neurocognitive functioning among PLWH. The primary outcome is physical activity while the secondary outcome is neurocognitive functioning. The preliminary results from the study [32] suggest that it is feasible to administer an SMS/MMS-mediated physical activity intervention to persons with HIV-associated neurocognitive disorders. Though the protocol of the study was not included in this review, it was documented because the recorded data from the future study will comprise part of the evidence from the literature that will guide practice.

The lack of reliable evidence to guide practice in this regard is an important gap in the literature because physical exercise has been found to minimize or improve co-morbidities associated with HIV/AIDS. Physical exercises improve cardiovascular fitness, muscle strength, lean body mass, instability of fat metabolism, increase bone mineral density, reduce risk of fracture and invariably enhance the quality of life in people living with HIV/AIDS [33, 34].

A previous study [35] conducted at the cardiovascular disease (CVD) prevention in Baltimore, Maryland, USA, examined the automated mHealth intervention for physical activity promotion and reported significant differential in activity levels. It was revealed that participants receiving mobile texts reminders recorded significant increases in their daily steps by 2534 (p < 0.001) compared to those who did not receive the SMS and by 3376 (p < 0.001) compared to blinded controls. For the secondary outcome, it was observed that participants in the unblinded-texts group compared to other groups recorded 23% increase in their total activity time or by 21 min/day and 160% increase in aerobic time or by 13 min/day, which was statistically significant. This should have implications for the overall wellbeing of participants because the World Health Organisation (WHO) reports that about 60% of people’s quality of life and health depends on their lifestyle and personal behaviour [34]. Therefore, if mobile texts could improve physical activity level in individuals, it would likewise improve their quality of life, and may also be applicable in PLWHA. An earlier narrative review of the literature [36] found that mobile text reminders resulted in improved treatment outcomes in 77% (46/60) of the studies, which may indicate its relevance in health care settings. Another review [37] of the literature found that among 16 RCTs, 10 reported significant improvement with the use of mobile phone text messaging in clinical and healthy behaviour interventions, and six RCTs reported differences suggesting positive trends. A systematic review [39] on the use of Mobile Apps and SMS Messaging as Physical and Mental Health Interventions showed improvement in physical health and significant reductions of anxiety, stress, and depression. Therefore, studies that investigate the effectiveness of mobile text reminders in improving physical exercise adherence among PLWHA may provide clinicians with relevant information that may improve the treatment outcome in this population.

### Quality of life

Only one study [21] which is of a high quality reported the effect of mobile text reminders on quality of life and found no significant improvement following SMS intervention. Thus, there is insufficient evidence from contemporary literature to draw a valid scientific conclusion on the estimate of effects. More RCTs are required to explore the benefits of improved ART and physical exercise adherence behaviours on QoL in PLWHA following SMS intervention. However, evidence from a recent systematic review of empirical studies in different patient populations [39] revealed that patients perceived mobile health or mHealth (including mobile apps, SMS text messaging, app combined with SMS text messaging) to be an effective treatment method. The systematic review found [39] significant, and positive improvements on health and well-being across the reviewed studies. Another systematic review [40] revealed that mobile health had a significant positive impact on attendance rates, clinical outcomes, chronic disease outcomes and health-related quality of life, and was cost-effective. Therefore, there could be translational benefits in adopting the same strategy in PLWHA which cannot be determined in our study considering the paucity of literature available in this area and highlights the need for future high-quality RCTs.

We identified four study protocols that evaluated QoL as one of the outcomes. One study protocol [41] aims to improve ART adherence and QoL of PLWH in China using mHealth (WeChat + SMS text message). It will further explore the feasibility and acceptability of the intervention approach in PLWH. The primary outcome measure is medication adherence while QoL is one of the secondary outcomes. Preliminary data from the pilot study showed no significant difference in the outcome measures between the groups. Another study protocol [42] seeks to determine the effects of WelTel Retain—weekly SMS communication (WelTel)—on keeping pre-ART patients in care. It further explores the cost-implications of the intervention. The study primary outcome will be 12-month retention in care while the secondary outcomes incremental cost-effectiveness ratio and QoL. Another protocol [43] seeks to determine whether a text messaging intervention (known as Connect4Care—C4C), meant to promote healthy behaviours, continual engagement with care, and antiretroviral persistence, will likewise improve viral suppression (primary outcome) in PLWH in San Francisco, USA. Some of the secondary outcomes include retention in care and QoL. A study protocol [44] aims to assess the impact of two-way and one-way SMS text messaging on adherence to HIV treatment in the Cameroonian population. The primary outcome is treatment adherence while the key secondary outcome is QoL. Although these protocols were not included in our review, it was important to highlight them considering that they will generate future data from RCTs on QoL outcomes in mhealth-mediated interventions.

High-quality RCTs in this area is important because the quality of life in PLWHA is an important factor that provides a basis for evaluating the impact of different health-related actions intended to improve wellbeing [45, 46]. However, it has socio-cultural dimensions and means different things to different people depending on their specific requirements, culture, goals, and expectations [47, 48]. QoL comprises multiple factors that in combination, add up to an individual sense of living well. For PLWHA, QoL is often considered as a good health indicator that provides insight on physical and mental well-being, functional independence, social relationship, and economic opportunities. Moreover, QoL assessment helps in making decisions about areas of need and the planning of interventions in the management of PLWHA [45]. These diverse definitions and perceptions of QoL across cultural communities would imply that the metrics for gauging this variable may likely vary across socio-cultural boundaries. Therefore, QoL cannot be determined and generalized based on a few studies otherwise it should be interpreted and related to the local context of the study. Overall, if mobile text reminders can improve adherence to medication as already seen in this study, then it should likewise have a positive effect on the QoL.

## Conclusions

### Implication for practice

Our review revealed promising evidence of the effectiveness of mobile text reminders in improving ART adherence behaviour in PLWHA. This is important because just as with other chronic conditions, adherence to prescribed medications have been the major problem of patients, and holds important significance for effective clinical management of PLWHA. Our review showed that the behavioural change models which informed the SMS reminders associated with positive outcomes were the theory of Planned Behavior and Starks 3-steps Adherence model. The common similarity of the above successful models is that they explore how an individual’s environment, including habitual behaviours, interacts with their health behaviour to inform the decision-making process to take a suggested action. Therefore, the above models could provide the clinician with an array of tools to develop and implement an SMS-driven behaviour change strategy to improve medication adherence. Since ART adherence behaviour is a healthy habit which has a direct impact on an individual’s health in chronic conditions [49] like HIV, the SMS reminder system should be prioritised at the initiation of ART in PLWHA. This agrees with a previous recommendation that on a diagnosis of a chronic health condition like HIV, major lifestyle changes need to be implemented [50, 51]. For HIV conditions, such major lifestyle changes include ART medication and physical exercise adherence.

The SMS reminder systems have been recommended as a potential mode of delivery in the clinical setting for behaviour change (adherence to medication/physical exercises) [52] likely to improve clinical outcomes (such as QoL) of PLWHA. Therefore, the SMS reminder systems could have comparable efficacy as well as complement educational meetings, educational detailing, and audit and feedback [53, 54] as important strategies for improved ART adherence behaviour in PLWHA. The studies included in this review reported several advantages of using SMS reminders that included ease of use, cheap services, and swift automated message delivery with negligible health risks. Though most participants found the reminders to be acceptable, one adverse event was reported [21] involving one female participant who felt that her undisclosed HIV status was compromised. Consequently, she requested to withdraw from the intervention arm of the study. Otherwise, no other undesirable effects have been reported across the studies. Considering the limitations of this review, especially the heterogeneity of the included studies, further investigation is required to generate a reliable estimate of the effectiveness of SMS reminder system in improving ART adherence behaviour to guiding practice. However, some studies in this review identified some logistic challenges associated with implementing the SMS strategy. The challenges are often encountered when deploying technological solutions in resource-limited settings and include less robust power supply and mobile phone infrastructure. The challenges could hinder the possibility of SMS reminder systems wholly benefitting from these solutions [23].

### Implication for research

The implications of the findings of our review suggest priorities for upcoming research that will address the remaining uncertainties we already highlighted around this area. Thus, while the results of our systematic review are promising, however, the observation of heterogeneity in the methods and outcome measures employed to evaluate the same treatment outcomes made it difficult to compare the effects of the intervention across multiple studies. Besides, the heterogeneity in the methods and outcome measures warrants a cautious interpretation of the findings. Also, it is not certain if these findings are of clinical significance because the effect size of the interventions was not provided in all the studies except one study [24] which showed a very small effect. Similarly, the only study on the effects of mobile text reminders on QoL included in this review reported no significant effects. However, the sample size of the study is underpowered to detect differences between the intervention group and the control group, and thus, its findings are unreliable. Therefore, there is a need for further evidence to conclude on the effectiveness of mobile text reminders in improving ART adherence behaviour in PLWHA.

Overall, there is limited evidence on the effectiveness of mobile text reminders in improving health outcomes such as QoL as well as lack of RCTs (evidence) on the effects of mobile text reminders on physical exercise adherence in PLWHA. These are important gaps in the literature that need to be addressed in future studies to guide practice. The observation of improved ART adherence behaviour in all the sufficiently powered studies included in this review emphasizes the effectiveness of mobile text reminders as behaviour change strategy and its relevance in HIV management. Therefore, it is recommended that:

i. Future studies should examine the effects of SMS frequency and timings; message content; optimal development processes, as well as identify and evaluate mechanisms by which text messages influence the behaviour change processes of the receivers.
ii. More adequately powered, good quality, randomised controlled trials should be conducted, particularly in both well-resourced and resource-limited settings.
iii. Trials of longer duration are also needed because in most cases there is a need for life-long adherence to treatments.
iv. Standardised outcome measures and approaches of measuring adherence (development of free and validated scores) are developed and used so that outcomes can be pooled across studies.
v. Since no review has examined how text messages are created, and if SMS is more effective when tailored to fit individual patient’s characteristics, and if some patients benefit more than others, it should be. Likewise, more researchers should consider conducting trials to address these areas.
vi. The development of mobile text messages should follow some theoretical framework, and text messages should be developed specifically for the target population and intervention.

### Limitation of study

The findings of this review might be limited for the following reasons: (1) the included studies were small, heterogeneous, and included participants not minding their adherence level, (2) Due to heterogeneity in the methods and outcome measure, it was difficult to conduct a meta-analysis of the included study. Therefore, this review cannot benefit from pooled estimates to determine evidence of effects across studies, (3) Inclusion of studies regardless of where the participants were recruited and enrolled, (4) Studies were not excluded based on how the text messages were developed, or if they were one way versus two ways, (5) Language restrictions were applied in this review, and thus may not reflect the totality of the evidence in this area since other studies published in other languages other than English were excluded, and finally, (6) Each study has at least one risk of bias domain judged as high risk.

## Supplementary Information

**Additional file 1.** Search strategy in PubMed for Medication adherence, Physical exercises adherence, quality of life.

## Data Availability

The datasets supporting the conclusions of this article are deposited in a public data repository—*figshare,* and are available using this link: https://figshare.com/s/88308033a10a271462a8. The DOI is: https://doi.org/10.6084/m9.figshare.15031782. All requests for the study data should be addressed to the corresponding author via email: sam.ibeneme@unn.edu.ng.

## Abbreviations

HIV: Human immune deficiency virus
AIDS: Acquired immune deficiency syndrome
ART: Antiretroviral therapy
HAART: Highly antiretroviral therapy
PLWHA: People living with HIV/AIDS
SMS: Short message service
QoL: Quality of Life
ROB: Risk of bias
RevMan: Review manager
RCTS: Randomised control trials
MeSH: Medical subject heading
AMED: Allied and complementary medicine database
CINAHL: Cumulative index to nursing and allied health literature
EMBASE: Excerpta medica database
AMED: Allied and complementary medicine database
PEDro: Physiotherapy evidence database
N/A: Not applicable
GRADE: Grading of recommendations assessment development and evaluation
INPLASY: International platform of registered systematic review and meta-analysis protocols
PRISMA: Preferred reporting items for systematic reviews and meta-analyses
HRQoL: Health-related quality of life
