# Supplementary material for: Effectiveness of mobile text reminder in improving adherence to medication, physical exercise, and quality of life in patients living with HIV: a systematic review
Source: BMC Infect Dis. 2021 Aug 23;21:859. doi: 10.1186/s12879-021-06563-0 (PMC8381579; doi:10.1186/s12879-021-06563-0)
Supplement: Supplementary file 1 — Additional file 1. Search strategy in PubMed for Medication adherence, Physical exercises adherence, quality of life. [file 12879_2021_6563_MOESM1_ESM.docx]

**Appendix I:** Search Strategy

| **CONCEPT** | **SEARCH TERMS** |
| --- | --- |
| **Population** | 1. HIV infections 2. HIV-1 3. HIV-2 4. Humans living with HIV 5. Adults living with HIV 6. Adolescence living with HIV 7. Aged HIV patients 8. HIV patients 9. People living with HIV 10. Human immunodeficiency syndrome 11. HIV long-term survivors 12. Seropositive HIV individuals 13. 1 OR 2 OR 3 OR 4 OR 5 OR 6 OR 7 OR 8 OR 9 OR 10 OR 11 OR12 |
| **Intervention** | 1. Cellphone 2. Cellular phone 3. Reminder systems 4. Text messaging 5. Transportable cellular phone 6. Cellular telephone 7. Portable cellular phone 8. Mobile phones 9. Cell phone use 10. Mobile cell reminder 11. Mobile cell reminder system 12. Messaging 13. Text 14. Short message service 15. SMS 16. Text message 17. Message 18. 14 OR 15 OR 16 OR 17 OR 18 OR 19 OR 20 OR 21 OR 22 OR 23 OR 24 OR 25 OR 26 OR 27 OR 28 OR 29 OR 30 |
| **Study design** | 1. Randomised controlled trial 2. Clinical trials 3. Random allocation 4. Control groups 5. 32 OR 33 OR 34 OR 35 |
| **Outcome** | 1. Quality of life 2. Medication adherence 3. Treatment adherence and compliance 4. Patients compliance 5. Exercise 6. Physical activity 7. Exercise adherence 8. Physical activity adherence 9. 37 OR 38 OR 39 OR 40 OR 41 OR 42 OR 43 OR 44 10. 13 AND 31 AND 36 AND 45 |
